# TRADD mediates the tumor necrosis factor-induced apoptosis of L929 cells in the absence of RIP3

**DOI:** 10.1038/s41598-017-16390-6

**Published:** 2017-11-23

**Authors:** Xixi Chang, Lili Wang, Zicheng Wang, Shuai Wu, Xiaoming Zhu, Shiping Hu, Yu Wang, Jiyun Yu, Guozhu Chen

**Affiliations:** Department of Frontier for Biological Treatment, Beijing Institute of Basic Medical Science, Beijing, 100850 China

## Abstract

Receptor-interacting protein kinase 3 (RIP3) is a critical initiator in mediating necroptosis induced by tumor necrosis factor alpha (TNFα) in L929 cells, so knockdown of RIP3 inhibits TNFα-induced L929 cell necroptosis. However, RIP3 knockdown was shown to switch TNFα-induced necroptosis to apoptosis in L929 cells in other studies. Therefore, whether RIP3 knockdown blocks the TNFα-induced death of L929 cells is controversial. In this study, TNFα activated caspase pathway and induced cell death in RIP3 knockdown L929 cells, and the RIP3-independent cell death had been blocked by Z-VAD-FMK (pan-caspase inhibitor) or caspase 8 knockdown, demonstrating that RIP3 knockdown switched TNFα-induced necroptosis to caspase-dependent apoptosis. Although both TNF receptor type 1-associated death domain protein (TRADD) and RIP1 have been reported to mediate TNFα-induced apoptosis, the knockdown of TRADD, but not RIP1, suppressed TNFα-induced activation of the caspase pathway and subsequent apoptosis in RIP3 knockdown L929 cells. In addition, TRADD bound and activated caspase 8 during the RIP3-independent apoptosis process, indicating that TRADD initiates RIP3-independent apoptosis by activating the caspase pathway. Collectively, we identified the target and mechanism underlying RIP3-independent apoptosis and elucidated the coordinated roles of RIP3 and TRADD in mediating the programmed cell death of L929 cells following TNFα stimulation.

## Introduction

Based on its morphological and biochemical features, programmed cell death has been classified into several distinct forms, including apoptosis, necroptosis and autophagic cell death^1,2^. A broad range of extracellular stimuli induce apoptosis and necroptosis, including death receptor ligation, Toll-like receptor ligands and virus infection^3–6^. In particular, necroptosis and apoptosis triggered by tumor necrosis factor alpha (TNFα) have been widely and intensively studied and documented^6–8^. TNFα is a pleiotropic inflammatory cytokine and plays important roles in multiple cellular functions, including cell proliferation, differentiation, apoptosis and necroptosis^9–11^. Upon ligation, TNF receptor 1 (TNFR1) recruits several adaptor/effector proteins bearing death domains (DDs) to form a TNFR1 signaling complex known as Complex I, which contains TNF receptor type 1-associated DEATH domain protein (TRADD), receptor-interacting protein 1 (RIP1), TNFR-associated factor 2 (TRAF2) and cellular inhibitor of apoptosis protein 1/2 (cIAP1/2)^10–13^. Complex I serves as a platform for the recruitment of downstream kinases and effector proteins to initiate the activation of the nuclear factor kappa B (NFκB) and mitogen-associated protein kinase (MAPK) pathways, leading to cell survival or proliferation^13–16^. In cells destined to die, TRADD and RIP1 dissociate from TNFR1 and recruit other proteins to form a secondary protein complex known as Complex II^14,15,17^. By recruiting the adaptor protein Fas-associated death domain (FADD) and pro-caspase 8, Complex II initiates apoptosis by activating the caspase pathway^16,18–20^. In contrast, in cells expressing high levels of receptor-interacting protein 3 (RIP3), RIP1 binds RIP3 to form a “necrosome” and then triggers necrotic cell death by activating the RIP1/RIP3 signaling pathway^8,17,21^. Therefore, the apoptotic and necroptotic processes induced by TNFα share some signaling pathways and adaptor/effector proteins^15,18,22,23^. However, TNFα usually induces necroptosis in cells in which apoptosis has been blocked by the caspase 8 inhibitor CrmA or the pan-caspase inhibitors Q-VD-OPH or Z-VAD-FMK (Z-VAD)^8,15,18^.

As a critical initiator of necroptosis, RIP3 is expressed at high levels in many different of cellular models of necroptosis, including L929 cells, and mediates TNFα-induced necroptosis by activating its substrate mixed lineage kinase domain-like protein (MLKL)^24,25^. Therefore, ectopic expression of RIP3 in HeLa or 3T3 cells promotes the activation of the necroptotic signaling pathway, resulting in a shift from TNFα-induced apoptosis to necroptosis^26,27^. Although RIP3 knockdown inhibits TNFα-induced necroptosis in L929 or mouse embryonic fibroblast (MEF) cells, it also has been reported to switch TNFα-induced necroptosis to apoptosis in L929 cells^26,28–30^. Therefore, the effect of RIP3 knockdown on TNFα-induced necroptosis in L929 cells is controversial. In addition, the exact target and detailed mechanisms involved in initiating the RIP3-independent cell death are unclear.

In the current study, we found that RIP3 knockdown switched TNFα-induced necroptosis to apoptosis in L929 cells. Moreover, TRADD, but not RIP1, was identified as the critical target protein in mediating RIP3-independent apoptosis by binding and activating caspase 8. Therefore, TRADD and RIP3 coordinately regulate signals required for programmed cell death triggered by TNFR1 ligation in L929 cells.

## Results

### RIP3 knockdown results in a shift from TNFα-induced necroptosis to apoptosis in L929 cells

**Figure 1:**
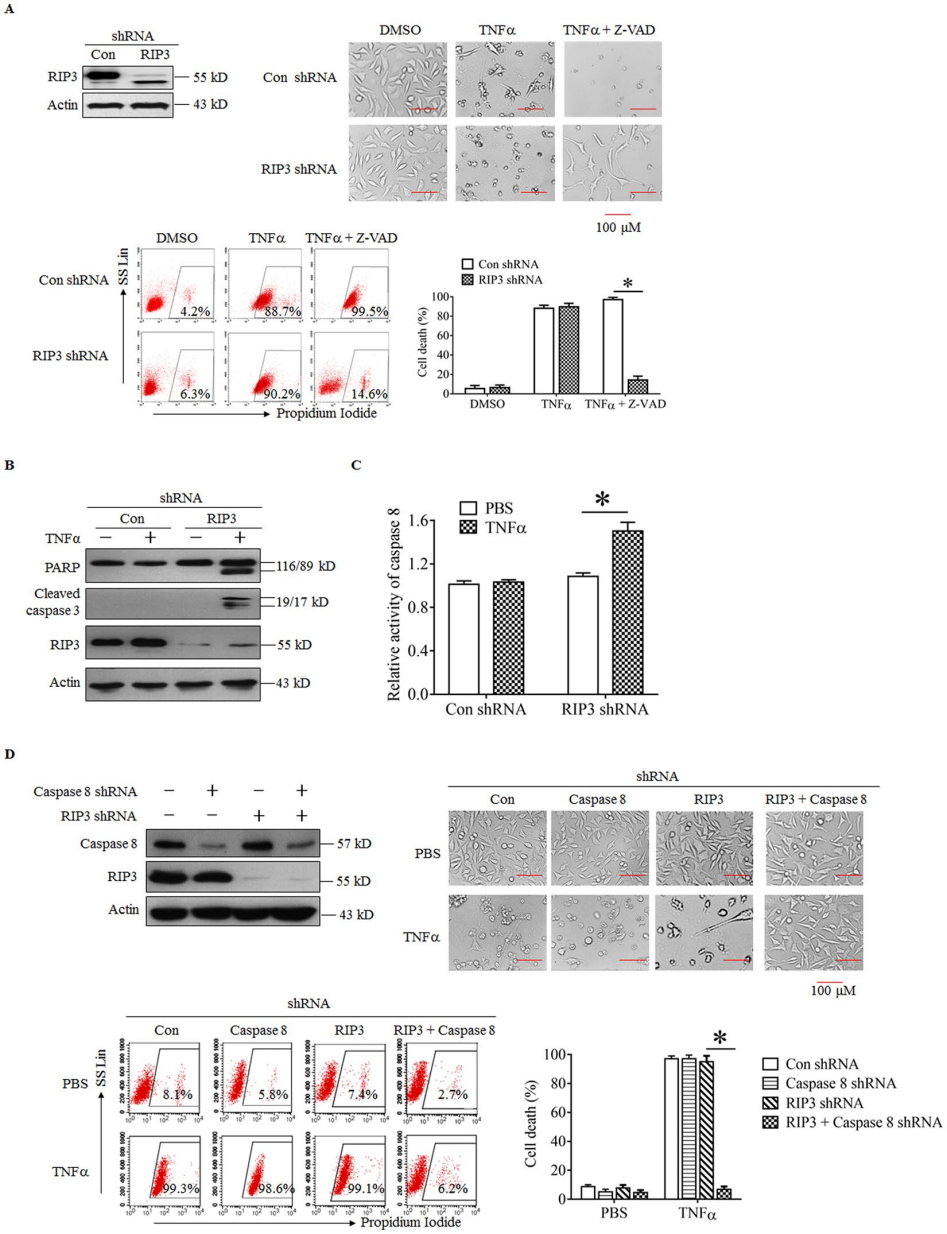
RIP3 knockdown switches TNFα-induced necroptosis to apoptosis in L929 cells. (**A**) Z-VAD blocks the TNFα-induced death of RIP3 knockdown L929 cells. The cells were infected with RIP3 shRNA or the control shRNA lentivirus, and western blotting was performed to determine the RIP3 knockdown efficiency. The full-length blots are presented in Supplementary Figure 1A. The cells were treated with TNFα or TNFα plus Z-VAD for 48 h, and cell death was measured by microscopy (200×) and flow cytometry. *P < 0.01. (**B**) RIP3 knockdown facilitates the TNFα-triggered activation of the caspase pathway. L929 cells were infected with the RIP3 shRNA or the negative control shRNA lentivirus and then treated with or without TNFα for an additional 12 h. Western blotting was performed to detect the knockdown efficiency and the cleavage of PARP and caspase 3. Actin was used as a loading control. The full-length blots are presented in Supplementary Figure 1B. (**C**) Caspase 8 activity was significantly increased in RIP3 knockdown L929 cells following TNFα stimulation. The RIP3 knockdown and negative control L929 cells were treated with or without TNFα for 12 h and then harvested to measure the activity of caspase 8. More than three independent experiments were performed for each group, and the relative activity of caspase 8 was calculated by normalizing the caspase 8 activity of all the groups with the activity of the negative control group. *P < 0.01. (**D**) Caspase 8 mediates the TNFα-induced death of RIP3 knockdown L929 cells. The knockdown of specific genes was mediated by infecting L929 cells with lentiviruses expressing shRNAs, and western blotting was used to evaluate the knockdown efficiency. The full-length blots are presented in Supplementary Figure 1D. The cells were treated with or without TNFα for 48 h, and cell death was measured by microscopy (200×) and flow cytometry. The RIP3 shRNA/DMSO and the RIP3 shRNA/TNFα FACS data prsented in Figure 1D are the same as that in Figure 2A. *P < 0.01.

Although RIP3 plays a critical role in initiating TNFα-induced necroptosis in L929 cells^8,17,21^. We found that RIP3 knockdown did not inhibit TNFα-induced L929 cell death (Fig. 1A). Moreover, Z-VAD, a pan-caspase inhibitor, almost completely blocked TNFα-induced cell death in RIP3 knockdown cells but not the negative control L929 cells (Fig. 1A), indicating that TNFα induces necroptosis in the negative control L929 cells but induces apoptosis in the RIP3 knockdown L929 cells. Therefore, RIP3 knockdown shifts TNFα-induced necroptosis to apoptosis in L929 cells. In addition, significant cleavage of caspase 3 and its substrate protein poly ADP ribose polymerase (PARP) was detected in RIP3 knockdown cells but not the negative control L929 cells following TNFα treatment (Fig. 1B), indicating that RIP3 knockdown facilitates activation of the caspase pathway. Moreover, caspase 8 activity was increased in RIP3 knockdown L929 cells but did not exhibit a significant change in the negative control L929 cells following TNFα stimulation (Fig. 1C), further confirming that RIP3 knockdown promotes the activation of the caspase pathway. Because caspase 8 is a key initiator of apoptosis induced by TNFR1 ligation, we determined the role of caspase 8 in the RIP3-independent cell death process. As shown in Fig. 1D, TNFα-induced RIP3-indpendent cell death was completely blocked by the simultaneous knockdown of caspase 8, further confirming that RIP3 knockdown promotes the death of L929 cells via the apoptotic pathway.

Based on these findings, RIP3 is the critical executor of TNFα-induced L929 cell necroptosis, and its downregulation switches TNFα-induced necroptosis to apoptosis.

### RIP1 does not mediate TNFα-induced apoptosis in RIP3 knockdown L929 cells

**Figure 2:**
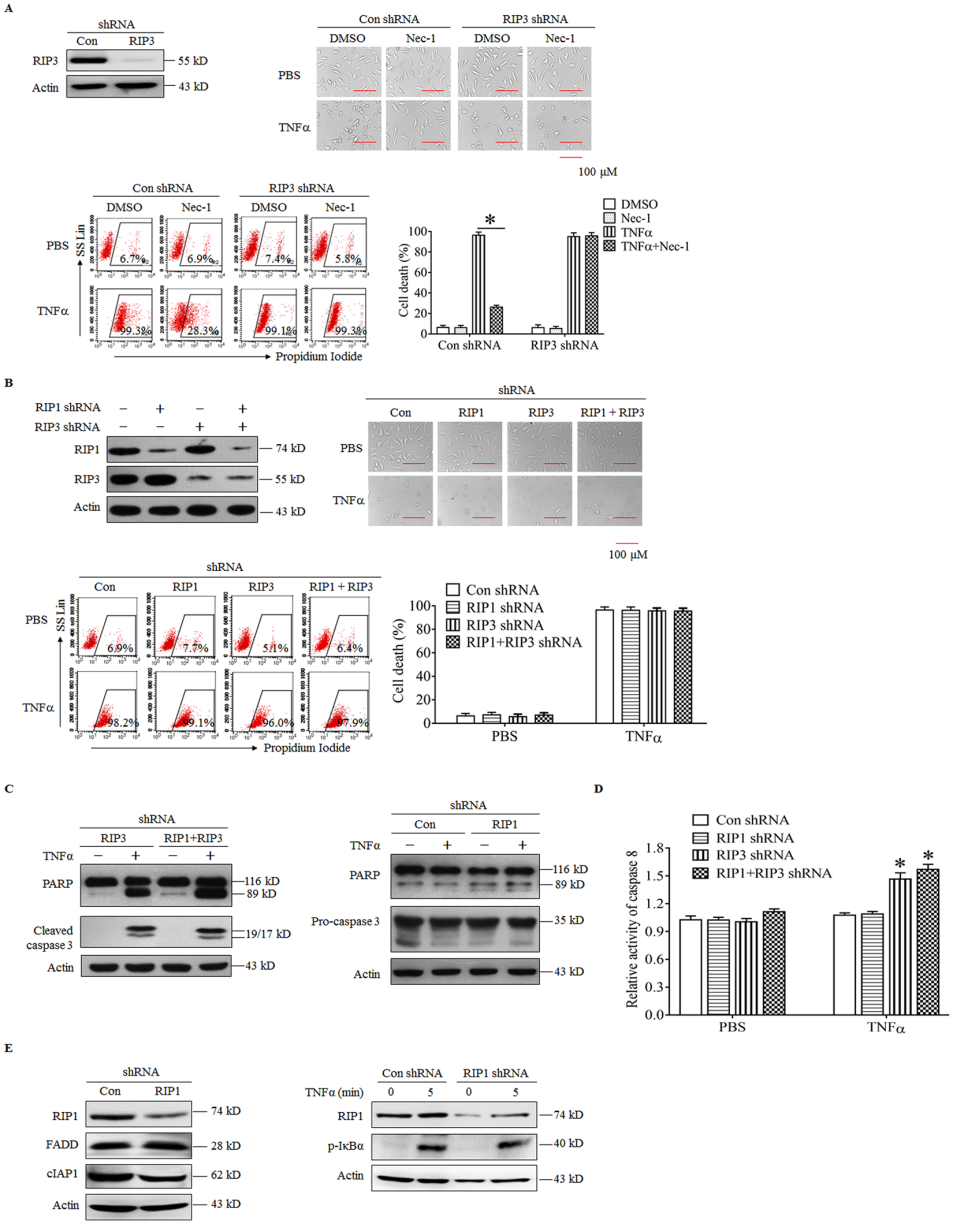
RIP1 does not mediate TNFα-induced apoptosis in RIP3 knockdown L929 cells. (**A**) Nec-1 does not block the TNFα-induced death of RIP3 knockdown L929 cells. The cells were infected with the RIP3 shRNA or the negative control shRNA lentivirus, and western blotting was performed to determine RIP3 knockdown efficiency. The full-length blots are presented in Supplementary Figure 2A. The cells were treated with TNFα or TNFα plus Nec-1 for 48 h, and cell death was measured using microscopy (200×) and flow cytometry. *P < 0.01 (**B**) RIP1 knockdown has no effect on TNFα-induced L929 cell death in the absence of RIP3. Knockdown of RIP1, RIP3 or RIP3 plus RIP1 was mediated by infecting L929 cells with lentiviruses expressing shRNAs, and western blotting was used to evaluate the knockdown efficiency. The full-length blots are presented in Supplementary Figure 2B. The cells were treated with or without TNFα for 48 h, and cell death was measured by microscopy (200×) and flow cytometry. (**C**) RIP1 knockdown has no inhibitory effect on the TNFα-triggered activation of the caspase pathway. RIP3 knockdown or RIP1 and RIP3 double-knockdown cells were treated with or without TNFα for 12 h, and western blotting was used to detect the cleavage of caspase 3 and PARP. Actin was used as a loading control. The full-length blots are presented in Supplementary Figure 2C. (**D**) RIP1 knockdown does not suppress caspase 8 activation. RIP1 knockdown, RIP3 knockdown or RIP1 and RIP3 double-knockdown L929 cells were treated with or without TNFα for 12 h and then harvested to measure caspase 8 activity. *P < 0.01 compared to the control shRNA group treated with TNFα. (**E**) The effect of RIP1 knockdown on FADD or cIAP1 protein level and NFκB pathway activation. RIP1 knockdown and negative control L929 cells were lyzed to determine the protein level of cIAP1 and FADD by using western blotting. Cells were also treated with or without TNFα for 5 minutes, and then lyzed to determine the level of IκBα phosphorylation by using western blotting. Actin was used as a loading control. The full-length blots are presented in Supplementary Figure 2E.

Both RIP1 and TRADD have been reported to mediate apoptosis induced by TNFR1 ligation^19^; therefore, we first determined the role of RIP1 in RIP3-independent apoptosis. As shown in Fig. 2A, necrostatin-1 (Nec-1), the allosteric inhibitor of RIP1, significantly inhibited TNFα-induced cell death in the control L929 cells but had no protective effects against cell death in RIP3 knockdown L929 cells following TNFα stimulation, indicating that RIP1 mediates TNFα-induced necroptosis in the negative control cells but not apoptosis in RIP3 knockdown L929 cells. In addition, we also found that TNFα-induced cell death in RIP3 knockdown L929 cells was not blocked by the simultaneous knockdown of RIP1 (Fig. 2B), further confirming the non-essential role of RIP1 in RIP3-independent apoptosis. Moreover, RIP1 knockdown did not block the cleavage of caspase 3 and PARP induced by TNFα in RIP3 knockdown L929 cells (Fig. 2C), and TNFα-induced increase in caspase 8 activity in RIP3 knockdown L929 cells was not suppressed by the simultaneous RIP1 knockdown (Fig. 2D). Therefore, knockdown of RIP1 did not suppress the TNFα-induced activation of the caspase pathway in RIP3 knockdown L929 cells, further confirming that RIP1 is not the target protein in mediating the RIP3-independent apoptosis induced by TNFα.

In addition, we found that RIP1 knockdown alone did not inhibit TNFα-induced L929 cell death, and no activation of caspase 8 and the subsequent caspase pathway had been observed during the process of cell death (Fig. 2B,C and D), indicating that TNFα induced RIP1-independent necroptosis in RIP1-knockdown L929 cells. We also detected the effect of RIP1 knockdown on the protein expression level of FADD and cIAP1, the two important proteins that mediate signal transduction initiated by TNFR1 ligation, and found that RIP1 knockdown had no effect on the protein level of FADD and cIAP1 (Fig. 2E). Moreover, RIP1 knockdown did not suppress the phosphorylation of IκBα in L929 cells following TNFα treatment (Fig. 2E), suggesting that RIP1 is not essential for the activation of NFκB pathway stimulated by TNFα.

Based on our data, RIP1 is not the target protein that initiates RIP3-independent apoptosis triggered by TNFα.

### TRADD mediates TNFα-induced apoptotic cell death in RIP3 knockdown L929 cells

**Figure 3:**
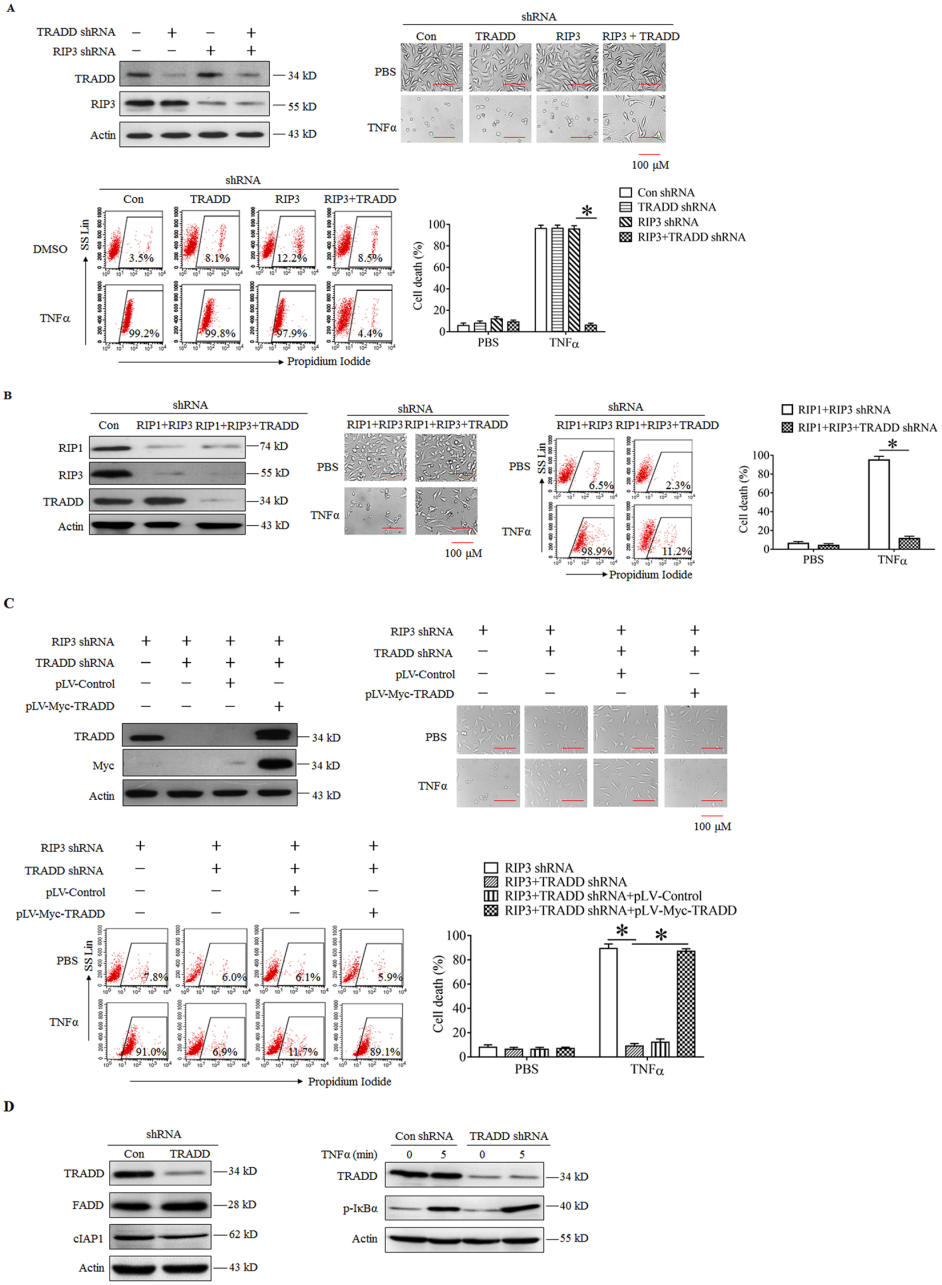
TRADD mediates TNFα-induced apoptosis in RIP3 knockdown L929 cells. (**A**) TRADD knockdown blocks the TNFα-induced death of RIP3 knockdown L929 cells. Cells were infected with the specific shRNA lentiviruses, and the knockdown efficiency was assessed by western blotting. The full-length blots are presented in Supplementary Figure 3A. The cells were treated with or without TNFα for 48 h, and cell death was measured by microscopy (200×) and flow cytometry. *P < 0.01. (**B**) TRADD initiates cell death in RIP1 and RIP3 double-knockdown L929 cells following TNFα treatment. RIP1 and RIP3 double-knockdown cells or RIP1, RIP3 and TRADD triple-knockdown L929 cells were generated by infecting cells with RIP1, RIP3 or TRADD shRNA lentiviruses in different combinations, and the knockdown efficiency was verified by western blotting. The full-length blots are presented in Supplementary Figure 3B. The cells were treated with or without TNFα for 48 h, and cell death was measured by microscopy (200×) and flow cytometry. *P < 0.01. (**C**) Restoration of TRADD expression restores the sensitivity of L929 cells to TNFα-induced cytotoxicity. L929 cells were infected with the indicated lentiviruses, and western blotting was performed to evaluate the expression levels of RIP3 and TRADD. The full-length blots are presented in Supplementary Figure 3C. The cells were treated with or without TNFα for 48 h, and cell death was measured by microscopy (200×) and flow cytometry. *P < 0.01. (**D**) The effect of TRADD knockdown on FADD or cIAP1 expression and the activation of NFκB signaling pathway. TRADD knockdown and the negative control L929 cells were lyzed to determine the protein level of cIAP1, FADD and TRADD by using western blotting. Cells were also treated with or without TNFα for 5 minutes, and the level of IκBα phosphorylation was detected by western blotting. Actin was used as a loading control. The full-length blots are presented in Supplementary Figure 3D.

Next, we explored whether TRADD mediated the TNFα-induced apoptosis of RIP3 knockdown L929 cells. As shown in Fig. 3A, though TRADD knockdown did not protect L929 cells from TNFα-induced necroptosis, it almost fully blocked TNFα-induced apoptosis of RIP3 knockdown L929 cells, indicating that TRADD is the target protein in initiating RIP3-independent apoptosis, but not RIP3-dependent necroptosis. In addition, we also explored the effects of TRADD on TNFα-triggered apoptosis in the absence of RIP1 and RIP3. As shown in Fig. 3B, TRADD knockdown prevented TNFα-induced apoptosis in L929 cells in which RIP1 and RIP3 had been depleted, confirming that TRADD plays a critical role in mediating apoptosis in the absence of RIP3 and independent of RIP1. Furthermore, we restored the expression of TRADD in RIP3 and TRADD double-knockdown cells by ectopically expressing Myc-tag TRADD and found that the Myc-tag TRADD was expressed at a level as high as endogenous TRADD (Fig. 3C). Moreover, the restoration of TRADD expression rescued the sensitivity of L929 cells to TNFα-induced cell death, further confirming the indispensable role of TRADD in mediating RIP3-independent apoptosis induced by TNFα. Finally, we determined the effect of TRADD knockdown on the FADD and cIAP1 expression and NFκB signaling pathway activation. As shown in Fig. 3D, TRADD knockdown had no effect on the protein expression level of FADD and cIAP1. Moreover, TRADD knockdown did not suppress the phosphorylation of IκBα induced by TNFα in L929 cells, indicating that TRADD are not essential for the activation of NFκB signaling pathway stimulated by TNFα.

In conclusion, our data demonstrate that TRADD is an essential target protein downstream of TNFR1 ligation just for initiating RIP3-independent apoptosis, but not necroptosis or NFκB pathway activation in L929 cells.

### TRADD mediates the RIP3-independent apoptosis through activating caspase signaling pathway

**Figure 4:**
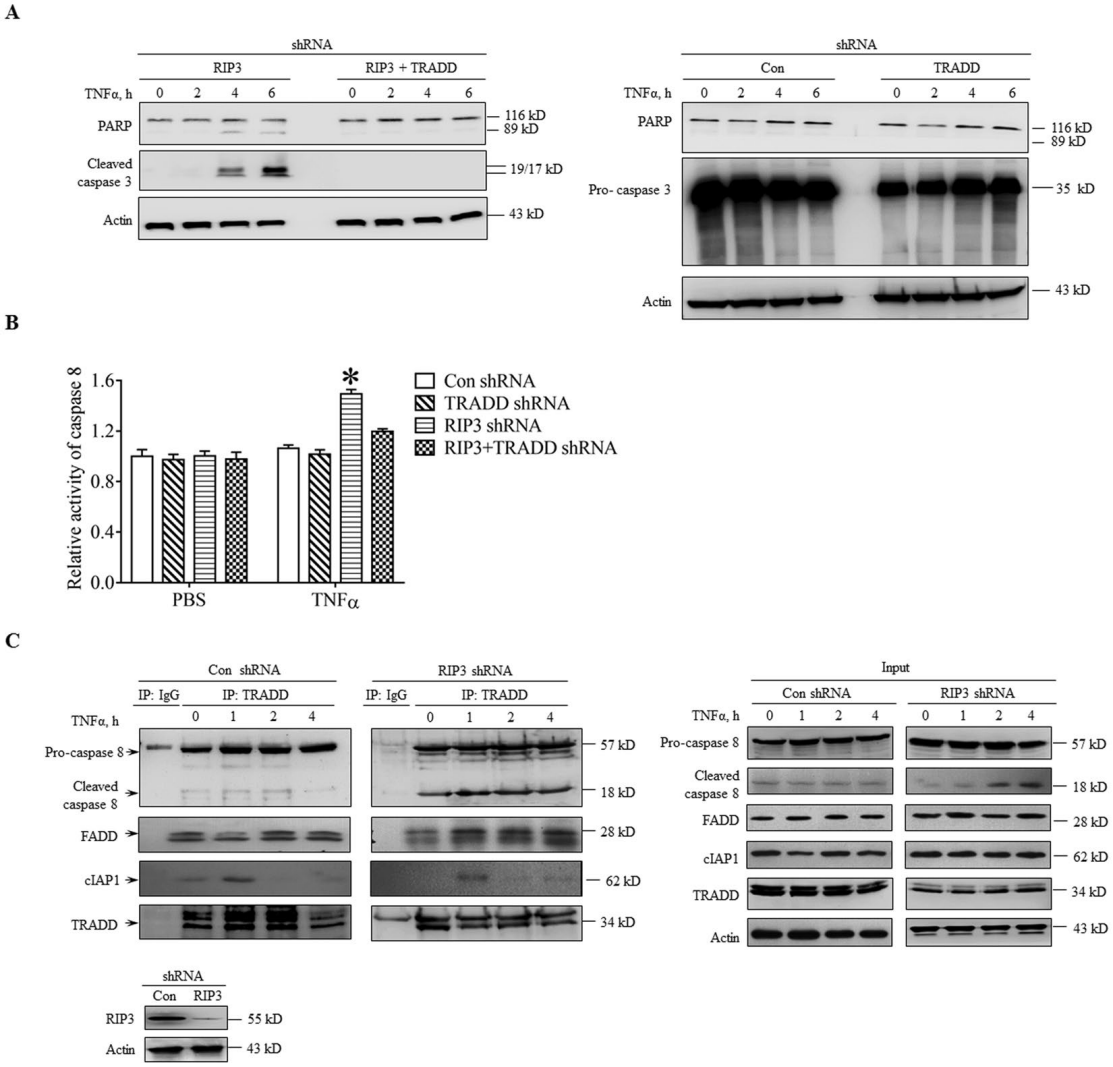
TRADD activates the caspase pathway by binding to and activating caspase 8. (**A**) TRADD mediates the TNFα-induced activation of the caspase pathway in the absence of RIP3. The negative control, TRADD knockdown, RIP3 knockdown or RIP3 and TRADD double-knockdown L929 cells were treated with TNFα for the indicated times, and the cleavage of PARP and caspase 3 was assessed by western blotting. Actin was used as a loading control. The full-length blots are presented in Supplementary Figure 4A. (**B**) TRADD knockdown suppresses TNFα-triggered caspase 8 activation in RIP3 knockdown L929 cells. TRADD knockdown, RIP3 knockdown or RIP3 and TRADD double-knockdown L929 cells were treated with or without TNFα for 12 h and then harvested for the measurement of caspase 8 activity. *P< 0.01 compared to the control shRNA group treated with TNFα. (**C**) RIP3 knockdown enhances the interactions between TRADD and caspase 8. RIP3 knockdown and the negative control L929 cells were treated with TNFα for the indicated times, and the cell lysates were immunoprecipitated with a TRADD antibody. Western blotting was used to detect TRADD, caspase 8, cIAP1, FADD and Actin. The full-length blots are presented in Supplementary Figure 4C.

It is well known that TNFα induces apoptosis through activating extrinsic caspase pathway, so we next determined the effect of TRADD knockdown on the activation of caspase pathway in RIP3 knockdown L929 cells following TNFα stimulation. As shown in Fig. 4A, significant cleavage of caspase 3 and PARP had been detected in RIP3 knockdown L929 cells, but not RIP3 and TRADD double knockdown L929 cells following TNFα treatment, indicating that TRADD is essential for the activation of caspase pathway induced by TNFα in RIP3 knockdown L929 cells. In addition, no cleavage of caspase 3 and PARP had been detected in TRADD knockdown and the negative control L929 cells following TNFα treatment (Fig. 4A), further confirming that TNFα induced necroptosis but not apoptosis in TRADD knockdown and the negative control L929 cells. We subsequently determined the role of TRADD in TNFα-induced caspase 8 activation in RIP3 knockdown L929 cells and found that TRADD knockdown significantly suppressed the TNFα-induced increase in caspase 8 activity (Fig. 4B), further confirming the critical role of TRADD in activating the caspase pathway during RIP3-independent apoptosis. Finally, we explored the interactions between TRADD and caspase 8 following TNFα treatment. As shown in Fig. 4C, TRADD was immunoprecipitated from L929 cells at various time points after TNFα treatment and pro-caspase 8 and cleaved caspase 8 (p18, the active form of caspase 8) were pulled down by TRADD. Although TRADD slightly interacted with pro-caspase 8 or cleaved caspase 8 prior to TNFα stimulation, the interaction between cleaved caspase 8 and TRADD was enhanced in RIP3 knockdown L929 cells, but not in control cells, as early as 1 h after TNFα treatment, indicating that RIP3 knockdown promotes interactions between TRADD and activated caspase 8 following TNFα stimulation. As the interaction between TRADD and caspase 8 is mediated by FADD, an adaptor protein can bind TRADD and caspase 8 directly, we next determined the protein level of FADD in the protein complex. As shown in Fig. 4C, the protein level of FADD in the protein complex slightly increased in RIP3 knockdown L929 cells in response to TNFα stimulation, further supporting the hypothesis that TRADD mediated apoptosis through initiating the activation of caspase pathway in the absence of RIP3.

In summary, TRADD mediates TNFα-induced apoptosis in RIP3 knockdown L929 cells by initiating activation of the extrinsic caspase pathway.

## Discussion

As the cross point during the necroptotic signaling pathway, RIP3 interacts with RIP1, TIR-domain-containing adapter-inducing interferon-β (TRIF) or Z-DNA binding protein 1 (DAI) to initiate necroptosis induced by death receptor ligation, Toll-like receptor ligation and virus infection, respectively^3,22,31^. RIP3 is expressed at high levels in L929 cells and initiates TNFα-induced necroptosis by activating its substrate protein MLKL; therefore, RIP3 knockdown or depletion blocks TNFα-induced L929 cell death in the absence or presence of Z-VAD^26,28,30,32^. However, our data demonstrated that RIP3 knockdown completely inhibited L929 cell death induced by TNFα plus Z-VAD, but not TNFα alone, indicating that Z-VAD suppressed TNFα-induced cell death in RIP3 knockdown L929 cells. Therefore, RIP3 knockdown switches TNFα-induced necroptosis to apoptosis in L929 cells. This finding was confirmed by our observation that the caspase signaling pathway is activated, and caspase 8 knockdown exerts a protective effect on RIP3 knockdown L929 cells stimulated with TNFα. Consistent with our results, RIP3 knockdown promotes a shift from TNFα-induced necroptosis to apoptosis in L929 sAhFas cells^29^. As shown in our previous study, TNFα induces necroptosis in L929-N cells but induces apoptosis in L929-A cells, and the RIP3 protein level is significantly higher in L929-N cells than that in L929-A cells^33^. The same results have also been observed in 3T3-A and 3T3-N cells following TNFα treatment^8^. Moreover, ectopic expression of RIP3 in MEF or HeLa cells switches TNFα-induced apoptosis to necroptosis^27,34,35^. Therefore, based on data from the current study and previous reports, the shift between necroptosis and apoptosis that occurs under certain cellular circumstances may depend on RIP3 protein levels.

Although RIP1 is a critical initiator of necroptosis, it also mediates apoptosis induced by TNFα and second mitochondria-derived activator of caspase (Smac) mimetics^14,19,36^. Moreover, in L929sAhFas cells, RIP3 knockdown also facilitates the shift from TNFα-induced necroptosis to RIP1-dependent apoptosis^29^. However, we found that repression of RIP1 by inhibition of kinase activity or gene knockdown had no effect on TNFα-induced apoptosis in RIP3 knockdown L929 cells. In addition, RIP1 knockdown did not suppress the TNFα-triggered activation of the caspase pathway; thus, our data demonstrated that RIP1 is not essential for the initiation of RIP3-independent apoptosis. The L929sAhFas cells used in the previous reports were produced by expressing the human Fas gene in L929sA cells, a TNFα-sensitive derivative of the murine fibrosarcoma cell line L929 cells^29,37^; therefore, some differences between these two cell lines may underlie the different functions of RIP1 in mediating RIP3-independent apoptosis. In addition, the differences between the methods used to downregulate the expression of RIP3 or RIP1 may be another reason for the differences observed in the ability of RIP1 to mediate RIP3-independent apoptosis. We used a specific shRNA expressed in a lentivirus to mediate gene knockdown, which results in stable repression of the expression of RIP3 and RIP1, but the knockdown of RIP1 or RIP3 mediated by transient transfection of a siRNA in the previous report lasts for very short period because the siRNA integrity is only efficiently maintained for 24 h after transfection.

TNFα is a pleiotropic cytokine that induces either apoptosis or proliferation^9,18^. Therefore, as the first adaptor protein identified to bind directly to the death domain of TNFR1, TRADD transduces the signals downstream of TNFR1, including the caspase, NFκB and MAP kinase pathways^10,38^. TRADD usually mediates TNFα-induced apoptosis in the presence of cycloheximide, which eliminates the endogenous inhibitor of apoptosis, FLICE inhibitory protein (FLIP), by suppressing gene expression promoted by activation of the NFκB pathway^39^. In this study, we found that TRADD knockdown inhibited TNFα-induced caspase activation and apoptosis in RIP3 knockdown L929 cells, and restoration of TRADD expression rescued the sensitivity of L929 cells to TNFα-induced cytotoxicity. Therefore, TRADD is the target protein required for mediating RIP3 independent apoptosis. In MEF cells, the recruitment of TRADD promotes the association of TNFR1 complex with FADD, which then binds and activates caspase 8, leading to apoptosis^11,13,39^. Consistent with the results from a previous study, we found that TRADD bound and activated caspase 8 in RIP3 knockdown L929 cells following TNFα stimulation, further conforming the critical role of TRADD in mediating the RIP3-indpendent apoptosis. Although TRADD and RIP1 have been reported to compete for binding to TNFR1, TRADD depletion completely blocks the recruitment of RIP1 to the TNFR1 complex in MEF cells or at least significantly weakens the interaction of RIP1 with TNFR1 in macrophages. Thus, RIP1 appears to require TRADD as an adaptor protein to indirectly associate with TNFR1^39–42^. Therefore, TRADD, but not RIP1, might transduce the apoptotic signal downstream of TNFR1 in RIP3 knockdown L929 cells. In addition, RIP1 usually mediates TNFα-induced apoptosis in the presence of Smac mimetics, which facilitates the release of RIP1 from the TNFR1 complex to form a new caspase 8-activating complex by promoting the degradation of RIP1 E3 ligase, cIAP1/2^19,43–45^. Therefore, TRADD and RIP1 mediate TNFα-induced apoptosis through different mechanisms, and TRADD is more likely to mediate TNFα-induced RIP3-independent apoptosis in the absence of Smac mimetics.

In summary, our study demonstrates that RIP3 knockdown switches TNFα-induced necroptosis to apoptosis in L929 cells. Moreover, TRADD, but not RIP1, initiates apoptosis by binding to and activating caspase 8 in RIP3 knockdown L929 cells following TNFα stimulation; therefore, TRADD and RIP3 coordinately mediate TNFα-induced programmed cell death in L929 cells.

## Materials and Methods

### Cells and reagents

L929 fibrosarcoma cells was obtained from the Cell Culture Center, Beijing Institute of Basic Medical Science of the Chinese Academy of Medical Science (Beijing, China). The cells were cultured in Dulbecco’s modified Eagle medium (Gibco, Grand Island, NY, USA) containing 10% fetal bovine serum (FBS, Kangyuan Biology, China). Necrostatin-1 (50 μM) and Z-VAD-FMK (20 μM) were purchased from Medchem Express (Beijing, China). TNFα (100 ng/mL) was obtained from GeneScript (Nanjing, China). propidium iodide (PI) was purchased from Sigma-Aldrich (St. Louis, MO, USA).

### Cell death analysis

Cell death was assessed by microscopy (200×), based on the presence of specific morphologic changes. Three fields in each group were observed, and representative images are shown. Cell death was also quantified by flow cytometry by measuring the ratio of PI-positive cells to the total number of cells. Briefly, the cells were collected by trypsinization and stained with PI. Then, cell death was assessed by flow cytometry (FACSCalibur, BD, USA) and CellQuest software (FACSCalibur, BD, USA). More than 10,000 cells were analyzed for each measurement. More than three independent experiments were performed in each group, and representative measurements are shown.

### Western blotting

For the western blot experiments, the cells were lysed in Laemmli buffer (Bio-Rad Laboratories, Hercules, CA, USA) and the protein concentration in the lysate was quantified by a BCA Protein Assay Kit (Pierce, Rockford, IL, USA). Sixty micrograms of total protein was loaded in each lane, and then the proteins were separated by SDS-polyacrylamide gel electrophoresis (SDS-PAGE) and electrically transferred to a polyvinylidene difluoride (PVDF) membrane (Sigma-Aldrich). After being blocked with 5% skim milk, the membrane was blotted with the appropriate primary antibodies for 12–16 h at 4 °C and then incubated with the appropriate horseradish peroxidase-conjugated secondary antibody (Zhongshan Biotechnology, Beijing, China) for 1–2 h at room temperature. The proteins were detected using the Tanon™ High-sig ECL Western Blot Substrate (Tanon Science & Technology, Shanghai, China), and digital images were obtained using a Gel-Imaging System (Tanon 5200, Shanghai, China). The following antibodies were used for the experiment: anti-RIP1 (610458, BD Transduction Laboratories, San Jose, CA, USA); anti-RIP3 (2283, ProSci, San Diego, CA, USA); anti-caspase 8 (ALX-804-447-C100, Enzo Lifescience, Lausen, Switzerland); anti-TNFR1 (Proteintech, Rosemont, IL, USA); anti-TRADD (sc-8436) and anti-FADD (sc-6036) (Santa Cruz, CA, USA); anti-cIAP1 (4952), anti-phopho-IκBα (2859), anti-PARP (9542), anti-caspase 3 (9665), anti-cleaved caspase 3 (9661) (Cell Signaling Technology, Beverly, MA, USA) and anti-β-actin (A5441) (Sigma-Aldrich).

### Immunoprecipitation

For the immunoprecipitation experiments, the cells were lysed in TL buffer containing 1% Triton X-100; 10% glycerol; 20 mM Hepes, pH 7.3; 150 mM NaCl; 5 mM EDTA; 5 mM NaF; 0.2 mM NaVO3 (Ortho); and complete protease inhibitor cocktail (Roche, Indianapolis) at 4 °C for 30 minutes. The cell lysates were subsequently centrifuged at 10,000 g for 10 min to remove cellular debris, and the protein concentration in the lysate was quantified with a BCA Protein Assay Kit (Pierce). Approximately 500 μg of total protein in the lysate was incubated with an isotype IgG control antibody (Zhongshan Biotechnology) and protein A/G Plus Agarose (Santa Cruz, CA) at 4 °C for 2 h for pre-cleaning. The pre-cleaned cell lysate was subsequently incubated with the appropriate primary antibody overnight at 4 °C. Immunoprecipitation was completed by adding protein A/G Plus Agarose (Santa Cruz) to the samples, incubating the samples for 2 h at 4 °C, and washing the protein A/G Plus Agarose three times with lysis buffer. Finally, the immunoprecipitants were denatured by the addition Laemmli buffer (Bio-Rad) and boiling for 5 min at 100 °C before being subjected to western blot analysis, as described above.

### Gene repression

For the gene repression experiments, the genes were downregulated via lentivirus transfection with specific shRNAs. The lentiviral vector pLKO.1-TRC was used to construct the shRNA vectors. The DNA fragments encoding shRNAs targeting specific genes or a non-specific gene (Con shRNA) were synthesized by Genewiz (Beijing, China) and inserted into the Age I and EcoR I site of the pLKO.1-TRC vector and verified by DNA sequencing. The newly constructed lenti-shRNA vectors were subsequently co-transfected into 293TN cells (System Biosciences, Mountain View, CA) with the indicated second-generation packaging systems (psPAX2 and pMD2.G vectors, Addgene, Cambridge, MA) using Chemifect transfection reagents (Fengrui Biotechnology, Beijing, China). The lentivirus-containing supernatant was harvested after 48–72 hours of transfection and filtered through a 0.22-μm filter. Transduction was performed in the presence of 10 μg/mL polybrene for 48–72 hours, and gene knockdown efficiency was verified by western blotting. DNA sequences targeting the following specific genes were inserted into the lenti-shRNA vectors:

mouse RIP1 (5′-GCATTGTCCTTTGGGCAAT-3′), mouse RIP3 (5′-GCTGAGTTGGTAGACAAGA-3′), mouse caspase 8 (5′-GAATGGAACCTGGTATATT-3′) and mouse TRADD (5′-GCAAAGACCCTCTAAGTACCCGGAC-3′).

### Caspase 8 activity assay

Caspase 8 activity was measured using the caspase 8 activity assay kit (Beyotime Institute of Biotechnology, Haimen, China) according to the manufacturer’s instruction. Briefly, cells were collected by trypsinization and lysed with lysis buffer. The protein concentrations in the lysates were quantitated with a Bradford assay kit (Bio-Rad). The lysates were mixed with the caspase 8 substrate (Ac-IETD-pNA) in a 96-well plate and then incubated at 37 °C for 30–120 min. The absorbance was measured at 405 nm and used to calculate caspase 8 activity. The relative activity of caspase 8 was calculated by normalizing the caspase 8 activity of each group with that of the normal control group.

### Ectopic expression of Myc-tagged TRADD

The Plv-Myc-mTRADD plasmid was purchased from Cyagen Biosciences (Guangzhou, China) and subsequently co-transfected into 293TN cells (System Biosciences) with the indicated second-generation packaging systems (psPAX2 and pMD2.G vectors) using Chemifect transfection reagents (Fengrui Biotechnology). The lentivirus-containing supernatant was harvested 48–72 h after transfection and filtered through a 0.22-μm filter. Transduction was performed in the presence of 10 μg/mL polybrene for 48–72 h, and the level of TRADD protein was verified by western blotting.

### Statistical analysis

GraphPad prism 5 software was used to analyze the data and construct statistical graphs. Statistical significance was analyzed using the unpaired t test and defined as P < 0.01. All the experiments were repeated at least three times, and the data are expressed as the mean ± standard deviation (SD) from representative experiments.

## Electronic supplementary material

Supplementary Information
