# Supplementary material for: TRADD mediates the tumor necrosis factor-induced apoptosis of L929 cells in the absence of RIP3
Source: Sci Rep. 2017 Nov 23;7:16111. doi: 10.1038/s41598-017-16390-6 (PMC5701027; doi:10.1038/s41598-017-16390-6)
Supplement: Supplementary file 1 — Supplementary Information [file 41598_2017_16390_MOESM1_ESM.pdf]

Title: TRADD mediates the tumor necrosis factor-induced apoptosis of L929 cells in the absence of RIP3.

Running title: TRADD mediates TNFα-induced apoptosis in RIP3 knockdown L929 cells.

Authors: Xixi Chang#, Lili Wang#, Zicheng Wang#, Shuai Wu#, Xiaoming Zhu#, Shiping Hu, Yu Wang*, Jiyun Yu*, Guozhu Chen*.

Department of Frontier for Biological Treatment, Beijing Institute of Basic Medical Science, Beijing,

100850, China.

#These authors contributed equally to this work and should be considered co-first authors.

Correspondences and requests for materials should be addressed to Guozhu Chen (E-mail: chenguozhu2002@126.com). The co-corresponding authors are Jiyun Yu (E-mail: yujyun@126.com) and Yu Wang (E-mail: 38290378@qq.com).

Figure legends

Figure 1. RIP3 knockdown switches TNFα-induced necroptosis to apoptosis in L929 cells.

(A). Z-VAD blocks the TNFα-induced death of RIP3 knockdown L929 cells. The cells were infected with RIP3 shRNA or the control shRNA lentivirus, and western blotting was performed to determine the RIP3 knockdown efficiency.

(B). RIP3 knockdown facilitates the TNFα-triggered activation of the caspase pathway. L929 cells were infected with the RIP3 shRNA or the negative control shRNA lentivirus and then treated with

or without TNFα for an additional 12 h. Western blotting was performed to detect the knockdown efficiency and the cleavage of PARP and caspase 3. Actin was used as a loading control.

(D). Caspase 8 mediates the TNFα-induced death of RIP3 knockdown L929 cells. The knockdown of specific genes was mediated by infecting L929 cells with lentiviruses expressing shRNAs, and western blotting was used to evaluate the knockdown efficiency.

Figure 2. RIP1 does not mediate TNFα-induced apoptosis in RIP3 knockdown L929 cells.

(A). Nec-1 does not block the TNFα-induced death of RIP3 knockdown L929 cells. The cells were infected with the RIP3 shRNA or the negative control shRNA lentivirus, and western blotting was performed to determine RIP3 knockdown efficiency.

(B). RIP1 knockdown has no effect on TNFα-induced L929 cell death in the absence of RIP3. Knockdown of RIP1, RIP3 or RIP3 plus RIP1 was mediated by infecting L929 cells with lentiviruses expressing shRNAs, and western blotting was used to evaluate the knockdown efficiency.

(C). RIP1 knockdown has no inhibitory effect on the TNFα-triggered activation of the caspase pathway. RIP3 knockdown or RIP1 and RIP3 double-knockdown cells were treated with or without TNFα for 12 h, and western blotting was used to detect the cleavage of caspase 3 and PARP. Actin was used as a loading control.

(E). The effect of RIP1 knockdown on FADD or cIAP1 protein level and NFκB pathway activation. RIP1 knockdown and negative control L929 cells were lyzed to determine the protein level of cIAP1 and FADD by using western blotting. Cells were also treated with or without TNFα for 5 minutes, and then lyzed to determine the level of IκBα phosphorylation by using western blotting. Actin was used as a loading control.

Figure 3. TRADD mediates TNFα-induced apoptosis in RIP3 knockdown L929 cells.

(A). TRADD knockdown blocks the TNFα-induced death of RIP3 knockdown L929 cells. Cells were infected with the specific shRNA lentiviruses, and the knockdown efficiency was assessed by western blotting.

(B). TRADD initiates cell death in RIP1 and RIP3 double-knockdown L929 cells following TNFα treatment. RIP1 and RIP3 double-knockdown cells or RIP1, RIP3 and TRADD triple-knockdown L929 cells were generated by infecting cells with RIP1, RIP3 or TRADD shRNA lentiviruses in different combinations, and the knockdown efficiency was verified by western blotting.

(C). Restoration of TRADD expression restores the sensitivity of L929 cells to TNFα-induced cytotoxicity. L929 cells were infected with the indicated lentiviruses, and western blotting was performed to evaluate the expression levels of RIP3 and TRADD.

(D). The effect of TRADD knockdown on FADD or cIAP1 expression and the activation of NFκB signaling pathway. TRADD knockdown and the negative control L929 cells were lyzed to determine the protein level of cIAP1, FADD and TRADD by using western blotting. Cells were also treated with or without TNFα for 5 minutes, and the level of IκBα phosphorylation was detected by western blotting. Actin was used as a loading control.

Figure 4. TRADD activates the caspase pathway by binding to and activating caspase 8.

(A). TRADD mediates the TNFα-induced activation of the caspase pathway in the absence of RIP3. The negative control, TRADD knockdown, RIP3 knockdown or RIP3 and TRADD double-knockdown L929 cells were treated with TNFα for the indicated times, and the cleavage of PARP and caspase 3 was assessed by western blotting. Actin was used as a loading control.

(C). RIP3 knockdown enhances the interactions between TRADD and caspase 8. RIP3 knockdown and the negative control L929 cells were treated with TNFα for the indicated times, and the cell lysates were immunoprecipitated with a TRADD antibody. Western blotting was used to detect TRADD, caspase 8, cIAP1, FADD and Actin.

The full length of blots mentioned in the manuscript has been listed as follows:

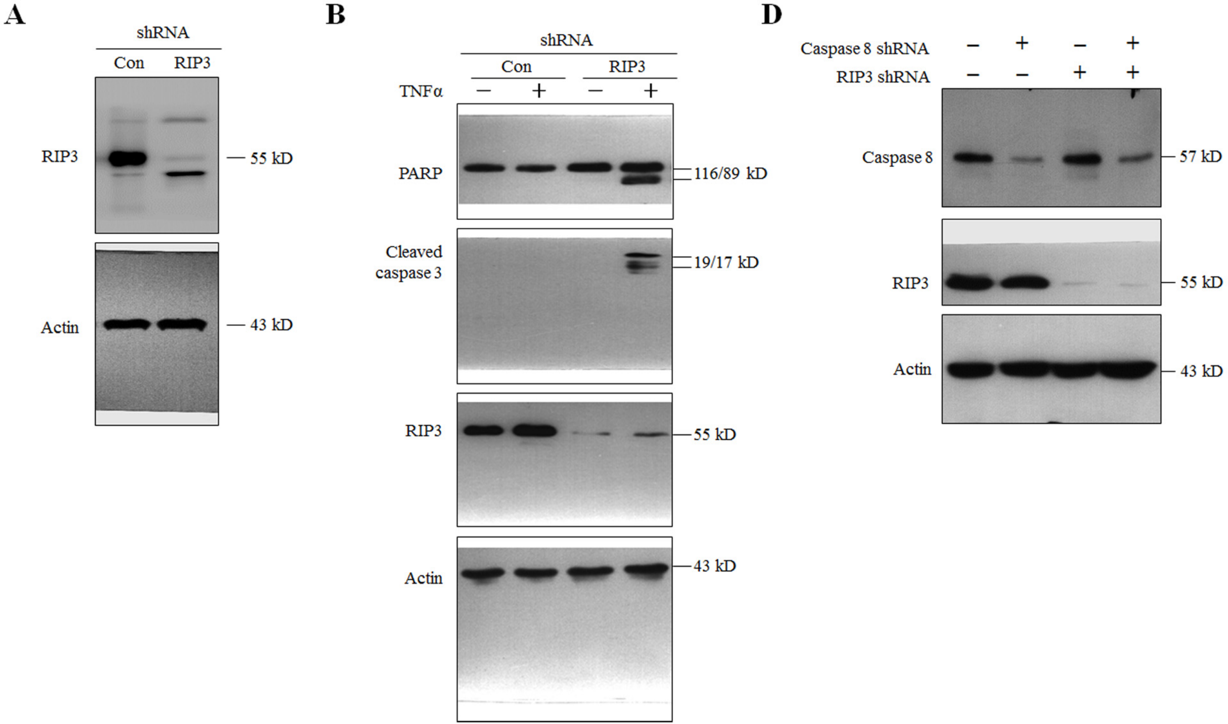
A
shRNA
Con
RIP3
RIP3
55 kD
Actin
43 kD
B
shRNA
Con
RIP3
TNFα
PARP
116/89 kD
Cleaved caspase 3
19/17 kD
RIP3
55 kD
Actin
43 kD
D
Caspase 8 shRNA
RIP3 shRNA
Caspase 8
57 kD
RIP3
55 kD
Actin
43 kD

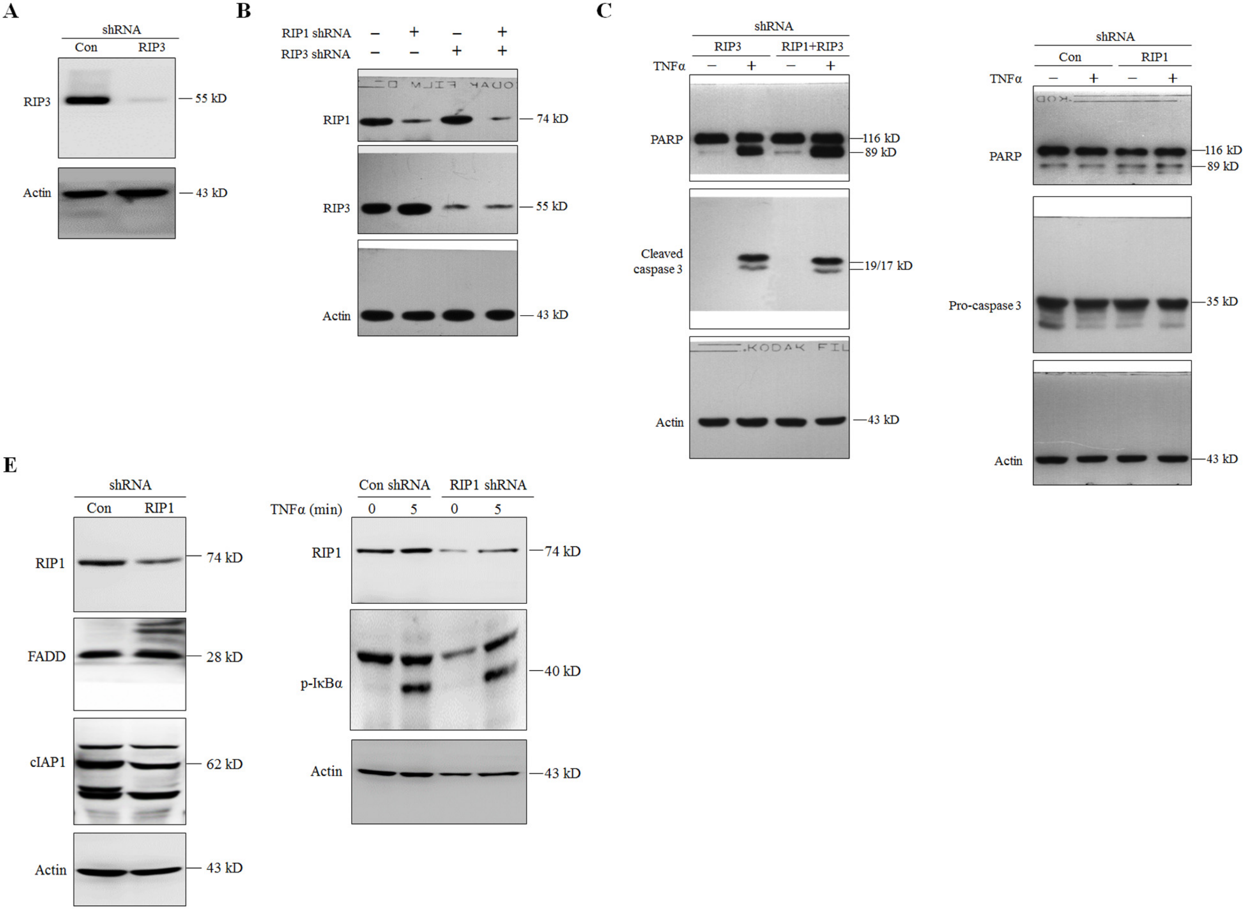
A
shRNA
Con
RIP3
RIP3
55 kD
Actin
43 kD
B
RIP1 shRNA
RIP3 shRNA
RIP1
74 kD
RIP3
55 kD
Actin
43 kD
C
shRNA
RIP3
RIP1+RIP3
TNFα
PARP
116 kD
89 kD
Cleaved caspase 3
19/17 kD
Actin
43 kD
shRNA
Con
RIP1
TNFα
PARP
116 kD
89 kD
Pro-caspase 3
35 kD
Actin
43 kD
E
shRNA
Con
RIP1
RIP1
74 kD
FADD
28 kD
cIAP1
62 kD
Actin
43 kD
Con shRNA
RIP1 shRNA
TNFα (min)
0
5
0
5
RIP1
74 kD
p-IκBα
40 kD
Actin
43 kD

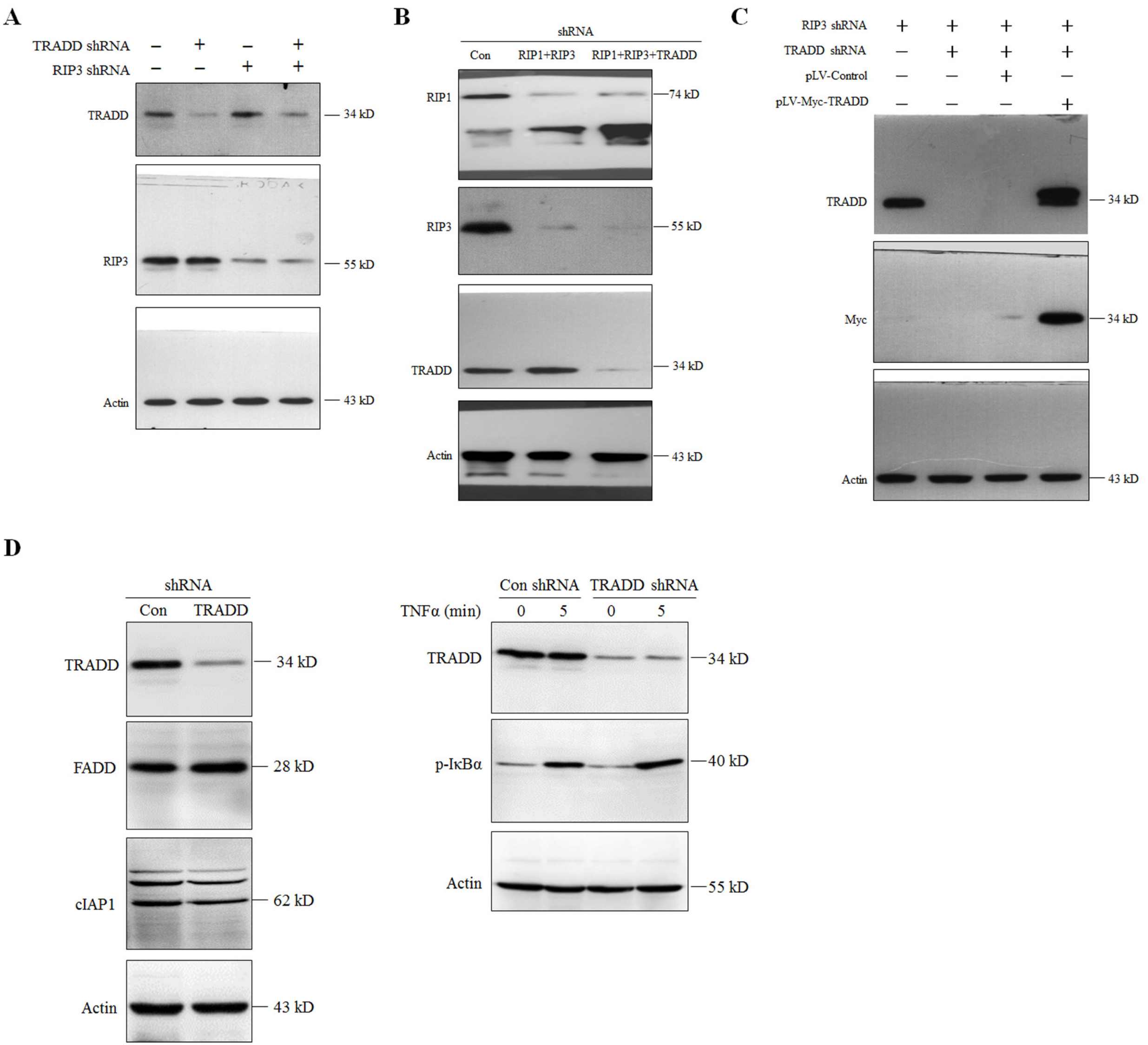
A
TRADD shRNA – + – +
RIP3 shRNA – – + +
TRADD
34 kD
RIP3
55 kD
Actin
43 kD
B
shRNA
Con
RIP1+RIP3
RIP1+RIP3+TRADD
RIP1
74 kD
RIP3
55 kD
TRADD
34 kD
Actin
43 kD
C
RIP3 shRNA + + + +
TRADD shRNA – + + +
pLV-Control – – + –
pLV-Myc-TRADD – – – +
TRADD
34 kD
Myc
34 kD
Actin
43 kD
D
shRNA
Con
TRADD
TRADD
34 kD
FADD
28 kD
cIAP1
62 kD
Actin
43 kD
Con shRNA
TRADD shRNA
TNFα (min) 0 5 0 5
TRADD
34 kD
p-IκBα
40 kD
Actin
55 kD

A

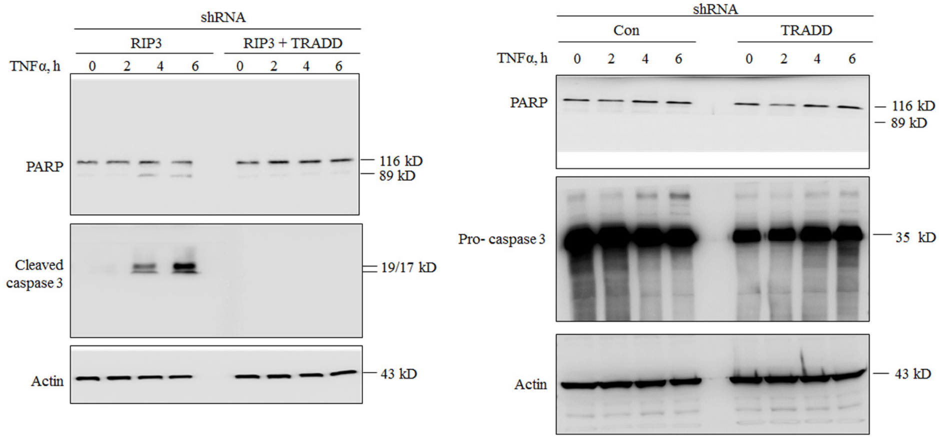
shRNA
RIP3
RIP3 + TRADD
TNFα, h
0 2 4 6 0 2 4 6
PARP
116 kD
89 kD
Cleaved caspase 3
19/17 kD
Actin
43 kD
shRNA
Con
TRADD
TNFα, h
0 2 4 6 0 2 4 6
PARP
116 kD
89 kD
Pro- caspase 3
35 kD
Actin
43 kD

C

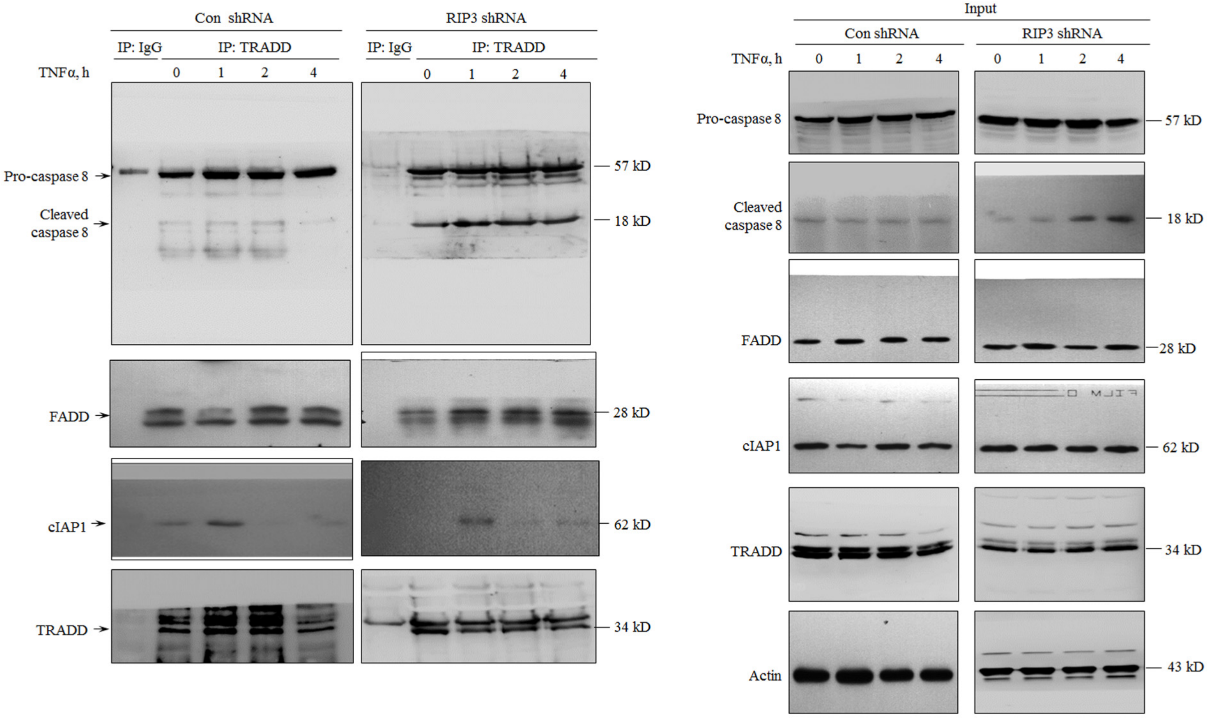
Con shRNA
RIP3 shRNA
IP: IgG
IP: TRADD
IP: IgG
IP: TRADD
TNFα, h
0 1 2 4
0 1 2 4
Pro-caspase 8
Cleaved caspase 8
57 kD
18 kD
FADD
28 kD
cIAP1
62 kD
TRADD
34 kD
Input
Con shRNA
RIP3 shRNA
TNFα, h
0 1 2 4 0 1 2 4
Pro-caspase 8
57 kD
Cleaved caspase 8
18 kD
FADD
28 kD
cIAP1
62 kD
TRADD
34 kD
Actin
43 kD

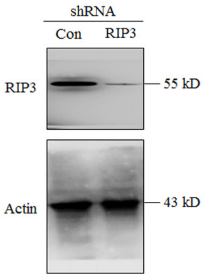
shRNA
Con
RIP3
RIP3
55 kD
Actin
43 kD
